# Potential Dupilumab‐Induced Unmasking of Specific Immunoglobulin Deficiency: A First‐of‐Its‐Kind Case

**DOI:** 10.1155/crpu/5248606

**Published:** 2026-05-23

**Authors:** Roshani Bista, Debbie Aishwarya Sathya, Sushan Gupta, Danish Thameem

**Affiliations:** ^1^ Department of Clinical Sciences, University of Illinois at Urbana-Champaign, Urbana, Illinois, USA, illinois.edu; ^2^ Division of Pulmonary and Critical Care Medicine, Medical College of Wisconsin, Madison, Wisconsin, USA, mcw.edu; ^3^ Division of Pulmonary and Critical Care Medicine, Carle Foundation Hospital, Urbana, Illinois, USA, carle.org

**Keywords:** dupilumab, hypogammaglobulinemia, IL-4 blockade, secondary immunodeficiency, specific antibody deficiency (SAD)

## Abstract

**Background:**

Dupilumab, a monoclonal antibody targeting the interleukin‐4 receptor alpha subunit (IL‐4R*α*), is approved for the treatment of various Th2‐mediated conditions such as atopic dermatitis, asthma, and chronic rhinosinusitis with nasal polyposis. While its side effect profile is well‐established, its impact on humoral immunity and potential to unmask subclinical immunodeficiencies remains underexplored.

**Case Presentation:**

We report a unique case of a 45‐year‐old woman with severe persistent asthma and chronic sinusitis who developed recurrent MRSA pneumonia and empyema after initiating dupilumab therapy. Initially treated with standard asthma therapies, she was started on dupilumab due to uncontrolled symptoms. One year into therapy, she developed a loculated empyema requiring surgical intervention. A second episode of MRSA empyema occurred 2 years later, necessitating further decortication. Her immunologic workup revealed a progressive decline in serum IgG levels (from 1100 to 851 mg/dL) and poor pneumococcal vaccine antibody responses, suggestive of impaired polysaccharide antibody response and possible specific antibody deficiency. Given the timing of immunologic decline in relation to dupilumab initiation and the known role of IL‐4 in B cell maturation and class‐switch recombination, we propose that IL‐4 blockade may have unmasked a previously subclinical immunoglobulin deficiency.

**Conclusion:**

Our case suggests a potential association between dupilumab therapy and unmasking of specific antibody deficiency. IL‐4 plays a critical role in B cell survival and immunoglobulin class switching; thus, its inhibition may impair humoral responses in susceptible individuals. Clinicians should maintain a high index of suspicion for immunodeficiency in patients on dupilumab who present with recurrent infections. Further research is warranted to elucidate the immunologic consequences of IL‐4 blockade and to identify patients who may be at risk for developing secondary immunodeficiency.

## 1. Introduction

Dupilumab, a monoclonal antibody targeting the IL‐4*α* receptor subunit, has emerged as a groundbreaking treatment for a range of conditions, including atopic dermatitis, asthma, COPD, chronic rhinosinusitis with nasal polyposis, and eosinophilic esophagitis. By inhibiting IL‐4 and IL‐13 signaling, dupilumab effectively modulates the Th2 inflammatory pathway, providing significant therapeutic benefits for patients with these chronic inflammatory diseases.

Side effects of dupilumab include conjunctivitis, keratitis, arthralgia, hypersensitivity reactions, and, in some cases, increased susceptibility to infections. However, the direct or indirect impact of dupilumab on humoral immunity specifically its potential role in unmasking or exacerbating immunodeficiencies has not been well explored in the literature.

Here, we present a unique case of dupilumab therapy potentially unmasking a previously undiagnosed immunodeficiency, highlighting a novel aspect of the drug’s immunological effects.

## 2. Clinical Course

A 45‐year‐old woman with a history of severe persistent asthma and chronic sinusitis presents to the pulmonology office for recurrent pulmonary infections. She was started on dupilumab a year ago due to uncontrolled asthma despite mometasone–formoterol therapy. After initiation of dupilumab, she developed right‐lung pneumonia with loculated empyema requiring total lung decortication. The bronchoscopy and intraoperative culture revealed methicillin‐resistant *Staphylococcus aureus* (MRSA), and she received an extended course of trimethoprim‐sulfamethoxazole. Dupilumab was continued initially; however, due to recurrent asthma exacerbations and worsening cough, dupilumab was switched to benralizumab.

A year later, the patient was admitted with bilateral pleural effusions (left > right) despite being treated for community‐acquired pneumonia with levofloxacin a week prior. The patient was started on empiric meropenem and vancomycin. A left‐sided chest tube was placed and was found to have a left‐sided parapneumonic effusion with complicated empyema. Computed tomography (CT) chest on the following day showed worsening left loculated pleural effusion with near‐complete left lung collapse requiring left‐sided video‐assisted thoracoscopic surgery (VATS) with decortication. After resolution of the effusion, chest tubes were removed but required repeat placement due to worsening effusion a day later. This time, intraoperative cultures again grew MRSA, and Karius Digital culture grew *Actinomyces* and *Rothia*. She was treated with a 6‐week course of vancomycin and amoxicillin‐clavulanic acid.

Given her recurrent bacterial infections and chronic sinus infections, she was referred to an allergy‐immunologist. Her serum aeroallergen panel was negative, but her allergy intradermal skin testing was positive for dust mites and molds. The patient’s immunologic workup is summarized in Tables 1 and 2. Patient’s IgA and IgM levels measured were within normal limits. Baseline tetanus antibody titers were normal, indicating intact T‐cell function. Patient’s IgG levels were low‐normal IgG. Although the patient’s IgG level before initiation of biologic therapy is unknown, subsequent measurements demonstrated a gradual decline in IgG level over time (from 1100 mg/dL in 2020 to 851 mg/dL in 2022). Baseline pneumococcal titers provided protection against only 12 of 23 *Strep pneumo* serotypes. Post‐PPSV23 vaccine titers showed protection against only 11 of 23 *Strep pneumo* serotypes and, in fact, showed a decline in all titers, indicating a suboptimal pneumococcal antibody response to PPSV23, suggesting specific antibody deficiency (SAD). Additionally, lymphocyte subset analysis demonstrated an elevated CD4/CD8 ratio and increased HLA‐DR expression, indicative of chronic immune activation. The patient was subsequently started on subcutaneous immunoglobulin replacement therapy, leading to a marked reduction in sinopulmonary infections.

**Table 1:** Immunological workup.

No	Lab component	Value	Reference value
1.	Serum IgG		700–1600 mg/dL
	(01/30/2020)	1100 mg/dL	
	(03/24/2022)	888 mg/dL	
	(08/23/2022)	851 mg/dL	

2.	Serum IgA		70–400 mg/dL
	(03/24/2022)	447 mg/dL	
	(08/23/2022)	449 mg/dL	
	(12/29/2022)	323.2 mg/dL	

3.	Serum IgM		40–230 mg/dL
	(03/24/2022)	50 mg/dL	
	(08/23/2022)	50 mg/dL	

4.	Tetanus IgG		≥ 0.1
	(03/24/2022)	0.78 IU/mL	
6.	Lymphocyte subpopulation analysis		
i.	CD3		
	Percentage	73%	60%–88%
	Absolute count	1719/cumm	661–1963/cumm

ii.	CD4		
	Percentage	60%	31%–64%
	Absolute count	1413/cumm	365–1294/cumm

iii.	CD8		
	Percentage	12%	12%–40%
	Absolute count	283/cumm	187–781/cumm

iv.	CD19		
	Percentage	10%	6%–25%
	Absolute count	236/cumm	86–488/cumm

v.	CD16CD56		
	Percentage	10%	5%–25%
	Absolute count	236/cumm	76–467/cumm

vi.	CD2		
	Percentage	79%	
	Absolute count	1860/cumm	

vii.	CD40		
	Percentage	9%	
	Absolute count	212/cumm	

viii.	HLA‐DR		
	Percentage	22%	
	Absolute count	518/cumm	

ix.	CD3 HLA‐DR		
	Percentage	4%	
	Absolute count	94/cumm	

x.	CD16		
	Percentage	10%	
	Absolute count	236/cumm	

xi.	TCR alpha/beta		
	Percentage	70%	
	Absolute count	1648/cumm	

xii.	TCR gamma/delta		
	Percentage	1%	
	Absolute count	24/cumm	

xiii.	HLA‐ABC		
	Percentage	100%	
	Absolute count	2355/cumm	

xiv.	Beta‐2 microglobulin		
	Percentage	100%	
	Absolute count	2355/cumm	
xv.	CD4/CD8 ratio	5.0	0.9–4.4

**Table 2:** Pneumococcal vaccine titers.

	Prevaccination	Postvaccination	Reference range
*S. pneumo* Type 1	3.1	0.0	> 2.3
*S. pneumo* Type 2	2.0	1.81	> 1.0
*S. pneumo* Type 3	< 0.4	0.22	> 1.8
*S. pneumo* Type 4	0.8	0.19	> 0.6
*S. pneumo* Type 5	3.9	0.34	> 10.7
*S. pneumo* Type 8	10.4	2.04	> 2.9
*S. pneumo* Type 9N	Unable to perform	0.67	> 9.2
*S. pneumo* Type 12F	< 0.4	0.33	> 0.6
*S. pneumo* Type 14	0.6	3.21	> 7.0
*S. pneumo* Type 17F	1.7	1.45	> 7.8
*S. pneumo* Type 19F	2.6	1.30	> 15.0
*S. pneumo* Type 20	0.5	6.35	> 1.3
*S. pneumo* Type 22F	4.4	0.92	> 7.2
*S. pneumo* Type 23F	4.1	0.14	> 0.0
*S. pneumo* Type 6B	12.7	2.37	> 4.7
*S. pneumo* Type 10A	1.1	1.81	> 2.9
*S. pneumo* Type 11A	0.5	1.77	> 2.4
*S. pneumo* Type 7F	1.1	0.46	> 3.2
*S. pneumo* Type 15B	18.6	3.38	> 3.3
*S. pneumo* Type 18C	0.9	1.29	> 3.3
*S. pneumo* Type 19A	9.5	Unable to perform	> 17.1
*S. pneumo* Type 9V	1.2	0.62	> 2.6
*S. pneumo* Type 33F	1.7	2.67	> 1.7

*Note:* Highlighted are significant titers. Either of the two following conditions would be consistent with a normal response to *Streptococcus pneumoniae* vaccination: Antibody concentrations greater than or equal to the reference value for at least 50%–70% of serotypes in either a pre‐ or postvaccination sample. Antibody concentrations increased by 2‐fold or greater for at least 50% of serotypes when comparing the pre‐ to the postvaccination results.

## 3. Discussion

The development of hypogammaglobulinemia in patients receiving biologic therapies has been documented, particularly with agents targeting B cell pathways. Although antigen‐specific IgG is produced by plasma cells, which do not express surface CD20, a subset of patients develops hypogammaglobulinemia on rituximab, which is a CD20‐specific monoclonal antibody. However, reports associating dupilumab with the unmasking of a SAD are limited. On the contrary, a notable case involved a patient with TTC7A deficiency who exhibited hypogammaglobulinemia and lymphopenia; dupilumab was successfully used to manage refractory pruritus in this context [1].

Dupilumab, an IgG4 human monoclonal antibody, binds to IL‐4 receptor and inhibits IL‐4 and IL‐13, thus downregulating the TH2 inflammation. IL‐4 is a key cytokine in B cell development, survival, and differentiation. It plays an essential role in immunoglobulin class‐switch recombination (CSR), guiding B cells to switch from producing IgM to other isotypes, including IgG1 and IgE in mice and IgG4 and IgE in humans [2, 3]. IL‐4 stimulates immature B cells (CD23−) to transition into transitional B cells (CD23+), like the effects of B cell‐activating factor (BAFF), thus supporting B cell survival and maturation. BAFF is known to promote the survival and maturation of B cells, but IL‐4’s role in this process has not been well understood until now. Some studies suggest possible synergistic effects of IL‐4 and BAFF [4]. Additionally, IL‐4 enhances B cell activation by upregulating MHC Class II, CD80, and CD86, which are critical for antigen presentation and subsequent immune responses [2].

In theory, inhibition of IL‐4 signaling could affect these survival pathways and potentially influence B cell maturation and antibody production. However, this mechanism remains speculative in the context of the present case, as no quantitative B cell abnormalities were demonstrated. Rather than generalized hypogammaglobulinemia, the findings in this patient are more consistent with impaired specific antibody responses, and it highlights the complexity of immune modulation in patients receiving such therapies.

Beyond CSR, IL‐4 also plays a role in early B cell survival and tolerance checkpoints. Normally, immature B cells expressing autoreactive B cell receptors (BCRs) undergo apoptosis (negative selection) to prevent autoimmunity. IL‐4 has been shown to protect transitional B cells from apoptosis by suppressing Bim, a proapoptotic protein that triggers B cell death when the BCR is strongly engaged [2, 4, 5]. Theoretically, this IL‐4‐mediated survival mechanism suggests that blocking IL‐4 could increase B cell apoptosis, reduce the mature B cell pool, and limit antibody‐producing plasma cells, thereby contributing to hypogammaglobulinemia [4]. Given these fundamental roles, blocking IL‐4 signaling with dupilumab may theoretically interfere with normal B cell function, potentially leading to dysregulated B cell maturation and reduced specific IgG production, which may manifest as secondary immunodeficiency.

SAD is a primary immunodeficiency disease characterized by recurrent sinopulmonary infections with normal IgG, IgM, and IgG with a diminished antibody response to polysaccharide antigens postvaccination. SAD is primarily diagnosed by evaluating the response of the pneumococcal IgG titers postvaccination. A normal humoral response is achieving > 50%–70% or a > 2‐fold response in the vaccine challenge.

Although our patient had normal IgA, IgM levels, and low‐normal IgG, the impaired vaccine response with progressive decline of IgG raised concerns for underlying immunodeficiency. She had received the PPSV23 vaccine three times previously, but her baseline pneumococcal titers were positive only to 12 of 23 *Strep pneumo* serotypes (52%). Despite a vaccination challenge with PPSV23, the patient exhibited protection against only 11 of 23 serotypes (47%), with all titers showing a significant drop, demonstrating an inability to mount a normal humoral response and ongoing development of immunodeficiency. The low‐normal IgG levels with ongoing reduction in IgG production upon initiation of dupilumab are consistent with the likely inhibition of IL‐4‐mediated signaling in CSR [2, 3]. The patient’s history of recurrent bacterial infections, including MRSA pneumonia and empyema, further supported an evolving immunodeficiency. Additionally, lymphocyte subset analysis demonstrated an elevated CD4/CD8 ratio and increased HLA‐DR expression, indicative of chronic immune activation, which is likely due to ongoing airway inflammation rather than a primary T‐cell defect and therefore does not directly explain the patient’s impaired humoral response.

Our patient had low‐normal IgG levels, normal IgM and IgA levels on dupilumab, with progressive decline and poor pneumococcal vaccine antibody responses, suggesting impaired polysaccharide antibody response, raising the possibility of SAD.

Although dupilumab therapy was hypothesized to have unmasked a SAD and recurrent infections in our patient, additional clinical factors might have contributed to her clinical states. Patient’s history of chronic sinusitis increased the risk of recurrent MRSA infections, both due to persistent MRSA colonization in the nasal cavity and recurrent lower airway seeding from postnasal drip. Her poorly controlled asthma further predisposed her to recurrent pulmonary infections through decreased mucociliary clearance and Type 2 airway inflammation. Moreover, her history of surgical decortication for empyema might have resulted in pleural and pulmonary scarring, increasing the susceptibility to develop recurrent MRSA pneumonia.

Given the critical role of IL‐4 in B cell maturation and CSR, the inhibition of IL‐4 signaling by dupilumab may have contributed to impaired B cell function, unmasking an underlying susceptibility to SAD and poor specific antibody production. However, the exact mechanisms remain speculative, and further research is necessary to elucidate this potential association. While routine immunoglobulin screening is not recommended for patients on dupilumab, clinicians should maintain a high index of suspicion for secondary immunodeficiencies in patients presenting with unexplained infectious complications.

## 4. Conclusion

This case raises questions about a potential link between IL‐4 blockade and dysregulated B cell maturation, leading to secondary antibody deficiency and increased infection risk. Further studies are warranted to explore the long‐term immunologic consequences of Th2 pathway inhibition and determine whether IL‐4 blockade affects B cell function or unmasks pre‐existing, subclinical immune dysfunction in susceptible individuals.

## Funding

No funding was received for this manuscript.

## Conflicts of Interest

The authors declare no conflicts of interest.

## Data Availability

All data generated or analyzed during this study are included in this published article. Due to patient privacy and confidentiality concerns, no additional data are publicly available. Deidentified data can be made available from the corresponding author upon reasonable request.
